# Endemic and emerging arboviral diseases of livestock in Nigeria: a review

**DOI:** 10.1186/s13071-018-2911-8

**Published:** 2018-06-07

**Authors:** Daniel Oluwayelu, Adebowale Adebiyi, Oyewale Tomori

**Affiliations:** 1 Department of Veterinary Microbiology, University of Ibadan, Ibadan, Oyo State Nigeria; 2 Centre for Control and Prevention of Zoonoses, University of Ibadan, Ibadan, Oyo State Nigeria; 3 Nigerian Academy of Science, Academy House, University of Lagos Campus, 8A Ransome Kuti Road, Akoka, Yaba, Lagos State Nigeria

**Keywords:** Arboviruses, Livestock, Endemic, Emerging, Nigeria

## Abstract

Arthropod-borne viruses (arboviruses) are the largest biologic group of vertebrate viruses and constitute important emerging infectious disease agents globally. Arthropod transmission provides a way for viruses to cross species barriers since the same arthropod may bite animals that rarely or never come into close contact in nature. In Nigeria, arboviruses have, over several decades, caused severe diseases in livestock resulting in great economic losses and, sometimes, infection of humans leading to morbidity and mortality. In the present review, a computerized search of existing literature was conducted using the Google search engine and PubMed electronic database to identify and review relevant publications on arboviral diseases of livestock in Nigeria. The keywords used were ‘arbovirus’, ‘arthropod-borne viral diseases’ or ‘livestock diseases’ and ‘Nigeria’ while the Boolean operator ‘OR’ was used to combine and narrow the searches. Additional information was obtained by searching the veterinary libraries for journals not listed in the database. The available publications were thereafter reviewed and findings qualitatively described. Our findings revealed that although there were several studies on arboviruses and the livestock diseases they cause in Nigeria, most of such reports were made four to six decades ago, with only a few reported recently. Consequently, the true economic and public health impact of these diseases are likely to be underestimated, mainly due to under-reporting or lack of awareness of them. Thus, it is essential to update information on arboviral diseases in Nigeria in order to increase awareness of the diseases and facilitate their prompt identification and reporting. The importance of routine surveillance for arbovirus livestock diseases and sentinel herd monitoring as basis for development of an early warning and alert system to prevent future outbreaks is discussed.

## Background

Arthropod-borne viruses (arboviruses) represent a considerable threat to human and animal health worldwide as several of them are found in different parts of the world, including Africa. More than five hundred arboviruses are known, of which about fifty cause diseases in domestic animals and many of the same cause zoonotic diseases [1]. Arboviruses pathogenic for animals belong to seven virus families: *Togaviridae*, *Flaviviridae*, *Bunyaviridae*, *Reoviridae*, *Rhabdoviridae*, *Orthomyxoviridae* and *Asfarviridae* (Table 1). They are transmitted to animals by five groups of hematophagous arthropods of the sub-phylum Chelicerata (Order Acarina, families Ixodidae and Argasidae *-* ticks) or members of the class Insecta: mosquitoes (family Culicidae), biting midges (family Ceratopogonidae), sand flies (subfamily Phlebotominae) and cimicid bugs (family Cimicidae) [1].

**Table 1:** Arboviruses pathogenic for domestic and wild mammals

Family	Species	Vector
*Togaviridae*	Eastern, Western and Venezuelan equine encephalitis viruses, Sindbis, Middelburg, Getah and Semliki Forest viruses	Mosquitoes (family Culicidae)
*Flaviviridae*	Yellow fever, Japanese encephalitis, Murray Valley encephalitis, West Nile, Usutu, Israel turkey meningoencephalitis, Tembusu and Wesselsbron viruses	Mosquitoes (family Culicidae)
	Tick-borne encephalitis, louping ill, Omsk hemorrhagic fever, Kyasanur Forest disease and Tyuleniy viruses	Ticks (family Ixodidae)
*Bunyaviridae*	Rift Valley fever, La Crosse, Snowshoe hare and Cache Valley viruses	Mosquitoes (family Culicidae)
	Nairobi sheep disease, Soldado and Bhanja viruses	Ticks (family Ixodidae/Argasidae)
	Main Drain, Akabane, Aino, Shuni and Schmallenberg viruses	Biting midges (family Ceratopogonidae)
*Reoviridae*	African horse sickness, Kasba, bluetongue, epizootic hemorrhagic disease of deer, Ibaraki, equine encephalosis, Peruvian horse sickness and Yunnan virus	Biting midges (family Ceratopogonidae)
*Rhabdoviridae*	Bovine ephemeral fever, vesicular stomatitis-Indiana, vesicular stomatitis-New Jersey, vesicular stomatitis-Alagoas, and Cocal viruses	Sand flies (subfamily Phlebotominae)/mosquito
*Orthomyxoviridae*	Thogoto virus	Ticks (family Ixodidae)
*Asfarviridae*	African swine fever virus	Ticks (family Argasidae)

Arboviruses have been isolated from vertebrates (man, domestic and wild animals) as well as from invertebrates (arthropods) [2, 3]. The arthropod vector acquires virus by feeding on the blood of viraemic animals; the ingested virus replicates in the tissues of the arthropod and can then be transmitted by bite to susceptible vertebrates [4]. Consequently, arthropod transmission provides a way for these viruses to cross species barriers, since the same arthropod may bite birds, reptiles and mammals that rarely or never come into close contact in nature [5]. Most arboviruses have restricted natural habitats in which specific receptive arthropod and vertebrate hosts are involved in the viral life-cycle. Vertebrate reservoir hosts are usually wild mammals, birds and/or domestic animals. The domestic animal species are, in most cases, infected incidentally for instance, by the geographical extension of a reservoir vertebrate host and/or a vector arthropod [6]. In addition, arboviruses that cause periodic epidemics have ecologically complex enzootic cycles which often involve arthropod and vertebrate hosts that are different from those involved in epidemic or epizootic cycles. Furthermore, these enzootic cycles are critical in dictating the magnitude of epidemics because they are poorly understood and inaccessible to effective control measures, thereby providing for the amplification of such viruses [5].

Several factors, including deforestation with development of new forest-farmland margins, primitive irrigation systems which pay little or no attention to arthropod control, and uncontrolled urbanization with vector populations breeding in accumulations of water and sewage, have been implicated in the intrusion of humans and domestic animals into new arthropod habitats [6, 7]. In addition, new routing of long-distance bird migrations brought about by new man-made water impoundments and increased long-distance livestock transportation, with potential for carriage of viruses and arthropods, cause exposure of domestic animals to new arthropods. These activities disturb the natural ecology and hence natural arbovirus life-cycles, and have been incriminated in the geographical spread or increased prevalence of diseases caused by these viruses [5].

In the last six decades, several arboviruses of livestock were reported in Nigeria, with some being detected in the 1950s to 1980s [8], but with no recent cases reported. On the contrary, some arboviruses of livestock were only recently reported for the first time in the country [9, 10]. Consequently, many animal health professionals in the livestock industry including veterinarians and animal health technologists, as well as students, may not be aware of their existence. Therefore, to increase awareness of these zoonotic diseases among health workers, there is a need to present updated information about the arboviruses and the diseases they cause in livestock in Nigeria. In the present review, a computerized search of existing literature was conducted using the Google search engine and PubMed and AGORA electronic databases to identify and download relevant publications on arboviruses of livestock in Nigeria. Additional information was obtained by searching the veterinary libraries for journals not listed in the database. A total of eighty-five publications on arboviral diseases of livestock and/or zoonotic arboviral diseases were considered, while those that focused only on arboviral diseases in humans were rejected. The available publications were thereafter reviewed and findings qualitatively described.

## Endemic arboviruses of livestock in Nigeria

During the last six decades, several arboviruses causing disease in livestock have been isolated or reported in Nigeria and some have acquired an endemic status in the animal population (Table 2). The recognition of arboviral diseases of veterinary importance in domestic animals and the isolation of their etiological agents can be attributed to factors such as establishment of virus laboratories, availability of trained veterinarians and allied professionals, improvement in disease surveillance strategies as well as use of modern, more rapid and more sensitive diagnostic techniques and equipment.

**Table 2:** Arboviruses of livestock isolated/reported in Nigeria 1950–2016

Virus	First report (country)	First isolation/report in Nigeria	Source	Reference
Bluetongue	1905 (South Africa)	1974	*Culicoides* spp., shrews (*Crocidura* spp.)	[22]
Rift Valley fever virus	1930 (Kenya)	1959	Sheep	[45]
West Nile fever virus	1937 (Uganda)	1959	Animals	[68]
		1973	Humans	[69]
African horse sickness	1900 (South Africa)	1971	Horse	[107]
Crimean-Congo haemorrhagic fever virus	1944–1945 (Crimea)	1970	Cattle, goats, hedgehogs, ticks	[133]
African swine fever virus	1910 (Kenya)	2001	Pigs	[144]
Kotonkan	1967 (Nigeria)	1967	*Culicoides* spp.	[150]
Bovine ephemeral fever virus	1907 (Rhodesia)	1973	Cattle	[159]
Wesselsbron virus	1955 (South Africa)	1968	Camel	[162]
Schmallenberg virus	2011 (Germany)	2015	Cattle	[9]
Akabane virus	1959 (Japan)	2016	Cattle, sheep	[10]

### Bluetongue virus

Bluetongue (BT) is an infectious, non-contagious arthropod-borne viral disease of ruminants which was first described in an outbreak in sheep in South Africa [11]. It is caused by the BT virus (BTV) which is almost exclusively transmitted by arthropods of the *Culicoides* spp. and is the prototype species of the genus *Orbivirus*, family *Reoviridae* [12]. BTV is notifiable to the World Organisation for Animal Health (OIE), as trade and movement restrictions that may cause severe economic losses for affected regions are commonly implicated in outbreaks. Currently, 27 BTV serotypes are recognized worldwide; the 27th serotype (BTV-27) is a novel virus recently discovered in asymptomatic goats in Corsica, France [13]. This serotype exists so far as three different variants, namely v01-v03 [14]. Other variants of the virus include BTV-XJ1407 and BTV-X ITL2015 detected in goats from China [15] and Sardinia, Italy [16], respectively. Further, two putative novel serotypes, BTV-28 and BTV-29 that were detected in a Capripox vaccine in the Middle East and in an alpaca in South Africa, respectively, have recently been suggested [13, 17]. The clinical signs of BT are often severe in infected sheep while in cattle, goats and camelids, BT infection is usually asymptomatic, although some clinical cases were observed in cattle during the BTV-8 outbreak that occurred in North Europe (reviewed in [14]).

Since the first report of BT in Nigeria in 1943 [18], the disease has become widespread among domestic ruminants in the country with epizootics reported at different times [19–21]. According to Fagbami & Ojeh [8], indigenous breeds of sheep seem to be relatively resistant as outbreaks of the disease that have occurred principally involved exotic breeds of sheep. Lee et al. [22], during routine virus surveillance, successfully isolated BT viruses from *Culicoides* spp. (serotypes 4, 6, 10 and 16) and shrews (*Crocidura* spp.) (serotype 7). In addition, at least 14 BTV serotypes have been reported to exist in Nigeria [23], with no cross-immunity between the various serotypes. Also, studies in the last four decades [24–30] have revealed the presence of moderate to high levels of BTV-specific antibodies in sheep, goats and cattle from southern and northern Nigeria, an indication that the disease is enzootic in the country. Several species of *Culicoides* that feed on domestic ruminants, such as *Culicoides imicola* and *C. milnei*, which have been incriminated as biological vectors of BT elsewhere, occur in large numbers in Nigeria [31, 32]. This may account for the widespread nature of the disease in the country.

### Rift Valley fever virus

Rift Valley fever (RVF) is a zoonotic, arthropod-borne viral disease important in domesticated ruminants [33]. This disease is characterized by high mortality rates in young animals and abortions in pregnant ruminants [33, 34]. Rift Valley fever can affect many species of animals including sheep, cattle, goats, buffalo, camels, monkeys as well as gray squirrels and other rodents [34, 35]. It is caused by the RVF virus (RVFV), an RNA virus in the genus *Phlebovirus*, family *Bunyaviridae* [36], that is primarily transmitted by mosquitoes of the *Aedes* and *Culex* spp. The virus appears to survive in the dried eggs of *Aedes* mosquitoes and epidemics are associated with hatching of the eggs during years of heavy rainfall and localized flooding [33, 37]. The primary amplifying hosts are sheep and cattle. Once it has been amplified in animals, RVFV can also be transmitted by other vectors, including many mosquito species and possibly other biting insects such as ticks and midges. In addition, animal products such as fresh, chilled and frozen meat, milk and milk products, wool, bones, skin and hides have been speculated to be potential sources of the virus [38]. The virus can also be transmitted *in utero* to the fetus [37].

RVF was first reported during an epizootic of the disease in sheep in Kenya [39], and since then there have been several reports of outbreaks elsewhere in Africa [40–44]. The virus was first isolated in Nigeria from the tissues of imported Merino sheep that died of the disease [45]. In 1970, Lee isolated three strains of the virus from *Culicoides* spp. and *Culex antennatus* mosquitoes collected at the University of Ibadan farm (Annual Report, Virus Research Laboratory, Ibadan 1970; reviewed in [8]). Subsequently, studies have shown serological evidence of RVFV in sheep, cattle, goats, horses and camels [46–49]. It is not yet clear whether the indigenous breeds of sheep are susceptible to overt Rift Valley fever. Experimental transmission studies revealed that although the West African dwarf (WAD) sheep developed high fever and exhibited viraemia, they were not susceptible to fatal illness [50]. However, Tomori [51] reported death and abortion in adult WAD ewes experimentally inoculated with the Nigerian indigenous virus strain.

Rift Valley fever virus has been recognized in Nigeria for about six decades; nevertheless, the present knowledge of the epizootiology suggests that the virus does not constitute a serious threat to the livestock industry in the country. According to Fagbami & Ojeh [8], the Nigerian strains of the virus may be apathogenic to the local breeds of sheep and may differ antigenically from the more virulent strains isolated elsewhere. Moreover, serological investigations [52] revealed that the indigenous isolates of RVFV are similar to the neurotropic Smithburn strain obtained from Uganda, which has also not been associated with severe animal disease in that country.

### West Nile virus

West Nile virus (WNV) is a zoonotic, mosquito-borne pathogen that belongs to the Japanese encephalitis virus serocomplex within the genus *Flavivirus*, family *Flaviviridae* [53]. It was first isolated in 1937 from the blood of a woman in the West Nile province of Uganda who had a mild febrile illness [54]. Since then it has been associated with sporadic and major outbreaks in humans and horses, as frequent outbreaks with increased proportion of neurological disease cases have been reported [55, 56]. The virus is maintained in an enzootic cycle between ornithophilic mosquitoes and birds by infected *Culex* spp. mosquitoes. Consequently, a number of wild birds are the main reservoir hosts in enzootic areas. Infected birds develop transient viraemia that allows transmission of the virus to feeding female mosquitoes [57]. WNV is associated with high morbidity and mortality in birds, horses and humans [58, 59], thereby constituting a major veterinary and public health concern. Following bites from WNV-infected female mosquitoes, infection is often unapparent or mild in humans but may produce severe and even fatal encephalitis in horses [57, 60, 61].

West Nile virus has a worldwide distribution throughout Africa, the Middle East, Asia, southern Europe, Australia and North America [57, 62]. Two distinct lineages of the virus have been revealed by phylogenetic analysis of the genomes of a number of strains; lineage 1 viruses have been isolated from the north eastern United States, Europe, Israel, Africa, India and Russia while lineage 2 viruses, which have previously been isolated only in sub-Saharan Africa and Madagascar [63], are now endemic also in Europe and have been reported in humans, birds and mosquitoes [64–67].

In Nigeria, despite numerous isolations of WNV from the animal population and the demonstration of WNV-specific antibodies in human sera [68], the virus was not isolated from humans until 1973 when Tomori and co-workers successfully recovered it from the blood and serum of three febrile children in Ibadan [69]. During the last three decades, there have been reports of detection of WNV antibodies in domestic animals and humans in Nigeria [70–75]. In addition, viral RNA was detected by reverse transcriptase-polymerase chain reaction in humans and mosquitoes [74], while reports of co-infection of WNV in malaria and typhoid fever patients [76] as well as in cases of undifferentiated febrile illness [77] have also been made. These reports underscore the need for consideration of West Nile fever in the differential diagnosis of febrile illnesses in Nigeria since its clinical presentation could be mistaken for malaria and typhoid fevers, which are endemic in the country.

### Usutu virus

Originally isolated in South Africa in 1959 from *Culex neavei* mosquitoes [78], Usutu virus (USUV) is a mosquito-borne flavivirus belonging to the Japanese encephalitis virus serocomplex [79]. It is regarded as a neglected emerging arbovirus in Europe and Africa [80], and has been detected in mosquitoes, animals and humans in countries of both continents [81–89]. The natural life-cycle of USUV involves mosquito-bird-mosquito cycles, in which ornithophilic mosquitoes (mainly *Culex* spp.) act as vectors and birds (mainly wild birds) as amplifying hosts. It has been demonstrated that multiple mosquito and avian species are involved in perpetuating the USUV life-cycle [90, 91]. In addition to *C. neavei*, USUV has also been isolated from other *Culex* species including *C. quinquefasciatus*, an anthropophilic vector that was directly implicated in the emergence of the virus in Europe, and from *Aedes* mosquitoes (reviewed in [92]). Generally, mosquitoes facilitate viral transmission to humans, horses, dogs, wild boars and rodents which may act as incidental hosts [83, 85, 88]. The isolation of USUV from bats in Germany was recently reported [87].

Since its first discovery in Africa, USUV had typically been isolated from mosquitoes and had never been associated with serious illness in mammals [79]. The virus had been isolated from human sera in Africa only on two occasions: from a man with fever and skin rash in Central African Republic in 1981 and from a 10-year-old child with fever and jaundice in Burkina Faso in 2004 [84]. In Nigeria, apart from the first report of USUV made over four decades ago during a serological investigation to determine antigenic relationships among eight Nigerian WNV isolates [81], there is scarcity of information on USUV infection. These authors showed that one of the WNV isolates was actually a strain of USUV. Conversely, in the last two decades, USUV has been demonstrated to be responsible for several outbreaks of overt disease in birds in Europe. Specifically, severe neurologic symptoms, often fatal, in wild and domestic birds were observed [93, 94]. Likewise, cases of severe encephalitis and USUV neuroinvasive infections of humans have been reported in different European countries [89, 95, 96].

Considering that USUV is an emerging WNV-related flavivirus which causes, in endemic areas, severe disease in humans, there is a need to confront the menace of USUV and WNV with similar preventive measures while adopting the One Health approach for investigation of human cases that involve animals and insects [89].

### African horse sickness virus

African horse sickness virus (AHSV) is a double-stranded RNA virus belonging to the genus *Orbivirus* of the family *Reoviridae*. It causes African horse sickness (AHS), an acute or sub-acute infectious, non-contagious viral disease of equids (horses, mules, donkeys and zebras) characterized by fever, cardiac and pulmonary manifestations, and high mortality in susceptible animals. The virus exists as nine immunologically distinct serotypes, all of which have been identified and considered enzootic in sub-Saharan Africa [97, 98]. Although the zebra is considered the natural host and main reservoir of AHSV in Africa, other equine species and their crossbreeds are susceptible to infection and, with the exception of donkeys, usually show clinical disease and high mortality [99]. Owing to the potential of this virus to cause widespread death and debilitating disease in naive equid populations, it is listed as a notifiable equine disease by the World Organization for Animal Health (OIE), which makes outbreaks of the disease compulsorily notifiable [100]. Transmission of AHSV occurs almost entirely through hematophagous arthropods, which act as biological vectors. Field and laboratory-based studies have implicated *Culicoides* biting midges (Diptera: Ceratopogonidae) as the primary vectors of AHSV. By far the most important species in the field transmission of the virus is *C. imicola* [101, 102], although mosquitoes such as *Anopheles stephensi* and *Aedes aegypti*, the camel tick (*Hyalomma dromedarii*), the brown dog tick (*Rhipicephalus sanguineus*) and biting flies such as *Stomoxys calcitrans* have also been shown to be possible vectors (reviewed in [102]).

AHS was first described in West Africa during the mid-nineteenth century, and by the turn of the twentieth century its cyclical appearance every 5–6 years was anecdotally correlated with extensions of the rainy season [102]. According to Parker et al. [103], AHSV activity often peaks during the latter part of the rainy season due to the increase in *Culicoides* vector population during that period. In Nigeria, cases of AHS were described and diagnosed clinically and by serological tests in the northern parts of the country between 1931 and 1967 [104–106]. However, since the first documented outbreak and subsequent isolation of the virus from a dead horse in Nigeria in 1970 [107], sporadic outbreaks of AHS have occurred in different regions of the country [108–111]. However, unlike previous outbreaks in which AHSV serotype 9 was implicated, Fasina et al. [112] reported the first isolation of a serotype 2 AHSV in the northern hemisphere from an outbreak in Lagos in 2007. Furthermore, Lazarus et al. [113] reported the detection of AHSV by real-time reverse transcription-polymerase chain reaction in tissue samples of a captive zebra that died in a game reserve in Bauchi, Nigeria. In addition, serologic studies in domestic animals have revealed the existence of AHSV antibodies (serotypes 4 and 9) in horses, donkeys, camels and dogs in Nigeria [110, 114–116].

### Crimean-Congo haemorrhagic fever virus

Crimean-Congo haemorrhagic fever (CCHF) is a fatal zoonotic tick-borne viral infection endemic in many countries in Africa, Asia, Europe and the Middle East [117, 118]. The CCHF virus (CCHFV) is an RNA virus belonging to the *Nairovirus* genus of the family *Bunyaviridae*, and is primarily transmitted by *Hyalomma* ticks, particularly *H. marginatum*, although more than 30 tick species have been shown to be capable of transmitting the virus between vertebrate hosts [119, 120]. The geographical range of this virus is the most extensive of the medically significant tick-borne viruses important to human health [121]. Human infection occurs through bites of or crushing infected ticks on bare skin with an open wound [122], by direct contact with animal blood or tissues [123], and by drinking unpasteurized milk from infected animals. Aerosol transmission and possible horizontal transmission from mother to child have also been reported [121, 124]. The majority of CCHF cases have occurred in people involved in the livestock industry, such as agricultural and slaughterhouse workers and veterinarians [125]. However, this virus causes unapparent infection or mild fever in cattle, sheep and goats with viraemia of sufficient intensity to infect adult ticks [126]. Nonetheless, infected livestock, particularly cattle, could provide virus for tick-borne transmission to highly susceptible humans and therefore play an important role in the epidemiology of the disease [127].

In West Africa, studies have revealed the presence of CCHFV in Niger [128], Mauritania [129], Senegal [130], Burkina Faso [131] and Mali [132]. However, apart from earlier reports of CCHFV made over three decades ago in domestic animals, wildlife, ticks and humans in Nigeria [133–136], and the recent reports of serological and virological evidence of the virus in humans in Borno State [49, 137], there is paucity of information on CCHFV and its impact in Nigeria, especially in domestic animals.

### African swine fever virus

African swine fever (ASF) is a highly infectious and contagious disease of domestic pigs caused by the African swine fever virus (ASFV) [138], which belongs to the genus *Asfivirus* in the family *Asfarviridae* [139]. The disease occurs in both domestic and wild pigs throughout sub-Saharan Africa and is transmitted through the bite of soft ticks (*Ornithodoros moubata*). Maintenance and transmission of ASFV involves the cycling of virus between these ticks and the free-living population of warthogs and bush pigs [140]. Mortality due to ASF can be as high as 100% in a naive population resulting in severe economic losses and socio-economic impact on production, trade and food security. Whereas ASF is currently considered enzootic in eastern and southern Africa, and the epidemiologic cycles of importance in many of the countries in these regions are well understood [141], little is known about the epidemiology of the infection in West Africa despite evidence of considerable spread of disease in this region in the late 1990s [142].

The first confirmed outbreak of ASF in Nigeria was reported in Lagos and Ogun States in September 1997. Between September 1997 and July 1998, other outbreaks of the disease were reported from the same states, and it was speculated that the virus was introduced through pigs from the Republic of Benin [143]. The isolation and molecular characterization of the ASFV strain involved in the outbreak was reported by Odemuyiwa et al. [144]. Subsequently, sporadic outbreaks have occurred in Nigeria with devastating impacts on subsistence and commercial piggery activities [140, 145–148]. In addition, Luther et al. [149] reported detection of viral DNA in a Nigerian bush pig (*Potamochoerus porcus*) for the first time, thus establishing a role for wild pigs in the epidemiology of the disease in Nigeria.

### Kotonkan virus

Kotonkan virus (KOTV) is a rhabdovirus first isolated in Nigeria from *Culicoides* spp. collected at the University of Ibadan Agricultural Farm in December 1967 and found to be antigenically related to Mokola virus, a member of the rabies serogroup [150]. The first association of this virus with animal disease was reported when an exotic breed of cattle imported into Nigeria came down with a debilitating illness characterized by fever, lameness, muscle soreness, anorexia, weight loss and recumbency [150]. Although natural clinical disease in the indigenous breed of cattle is rare, experimental studies using a mouse brain-adapted strain of the virus showed that it was pathogenic for White Fulani cattle [151]. It has been suggested that the rarity of natural overt disease in the indigenous breeds of cattle may be due to the fact that young calves get infected when they still have some level of protective maternal antibody [150]. Although Kotonkan virus is not frequently encountered, serological studies have revealed that it is active in the country with neutralizing antibodies demonstrated in man and several species of domestic and wild animals [150].

### Bovine ephemeral fever virus

Bovine ephemeral fever virus (BEFV) is an arthropod-borne rhabdovirus which is classified as the type species of the genus *Ephemerovirus*. It causes an acute febrile illness of cattle and water buffalo known as bovine ephemeral fever (or three-day sickness, bovine enzootic fever, bovine influenza or stiffseitke) that is characterized by biphasic fever, salivation, oculo-nasal discharge, anorexia, recumbency, muscle stiffness, shifting lameness, and enlargement of peripheral lymph nodes [152]. A unique feature of the disease is its rapid onset and rapid recovery, lasting only 1–3 days, hence the name “three-day sickness”. Although BEF has been recognized for many decades in Africa [153], it is presently enzootic and seasonally epizootic in Australia, Asia, Africa and the Middle East, usually not extending beyond a zone limited by the latitudes of 38°N to 36°S [154, 155]. The disease produces considerable economic impact due primarily to cessation of lactation in dairy cattle, loss of condition in beef cattle and the immobilisation of water buffalo used for draught power [155, 156]. BEF also impacts on trade in live cattle from infected zones and there is evidence that the risks of inter-continental spread of BEFV through animal transport or vector translocation may be increasing [157]. BEFV has been isolated from various species of *Culicoides* including *C. imicola*, *C. pallidipennis* and *C. brevitarsis*, as well as from different mosquito species such as *Anopheles*, *Culex*, *Aedes* and *Uranotaenia* [reviewed in 152]. This rhabdovirus is also pathogenic for several laboratory hosts such as mice and cell culture [158]. The first virological evidence of BEF in Nigeria was in 1973, when two isolates of the virus were obtained from sick cattle on the University of Ibadan farm [159]. This was followed by the study of Tomori et al. [160], who demonstrated the presence of serum antibodies to the virus in calves. According to Kemp et al. [159], the disease has been known to herdsmen in Nigeria for many years, occurring regularly at the beginning of the wet season.

### Wesselsbron virus

Wesselsbron virus (WSLV), a mosquito-borne flavivirus, is the aetiological agent of a severe disease of sheep characterized by high fever, severe leucopenia in adult sheep, abortion in pregnant ewes and high mortality in lambs. The virus was originally isolated during a severe epizootic in sheep in South Africa [161] but was first isolated in Nigeria from the blood of an apparently healthy camel in 1968 [162]. Since then, no report of clinical cases of Wesselsbron virus infection has been made in the country. However, experimental studies have revealed that the virus can produce overt clinical disease in West African dwarf sheep [163]. Since its first isolation by Kemp et al. [162], the virus has not been isolated again from animals in Nigeria. This has been partly attributed to the low level of virus surveillance in domestic animals. In addition, the range system of animal husbandry in Nigeria and the occurrence of several flaviviruses (such as Yellow fever, West Nile and Dengue) in the country predispose the local livestock to mixed or multiple flavivirus contacts resulting in the development of heterologous flavivirus antibodies in domestic animals which probably modify the virus activity [8]. Such immunity has been shown to reduce both the level of viraemia and severity of clinical disease in indigenous breeds [164]. However, the virus has been successfully isolated from humans [165], while antibodies against it have been demonstrated in animals [71, 166, 167].

## Emerging arboviruses of livestock in Nigeria

An emerging viral disease is one that is newly recognized or newly evolved, or that has occurred previously but shows an increase in incidence or expansion in geographical, host, or vector range [5]. In recent years, some diseases of livestock caused by arboviruses such as Schmallenberg virus and Akabane virus that have hitherto not been reported in Nigeria have been described. It is therefore important that field veterinarians, animal health workers and undergraduate students of Veterinary Medicine in the country be aware of their existence.

### Schmallenberg virus

Schmallenberg virus (SBV) was first detected in November 2011 in a pool of blood samples from clinically affected dairy cows following reports of hyperthermia and drop in milk production in adult dairy cows in North-west Germany and The Netherlands [168]. The virus is a novel member of the family *Bunyaviridae*, genus *Orthobunyavirus* and is an enveloped, negative-sense, segmented, single-stranded RNA virus that is closely related to Shamonda virus, which belongs to the Simbu serogroup of viruses that includes Sango, Sabo and Shuni viruses [169, 170]. Since the first cases from Germany and The Netherlands, this economically important disease in ruminants has caused a large epidemic with several reports of clinical and serologic evidence of the disease mostly from Europe [171–173]. SBV is associated with abortions, stillbirths, and congenital malformations in cattle, sheep and less often, goats [174] and has been shown to be neurotropic in lambs and calves infected *in utero* [175, 176]. The virus is also able to infect wild cervids and llamas but no clinical signs or macroscopic abnormalities have been recorded for these species [177, 178].

Several studies have reported the presence of SBV in different species of *Culicoides* which has been identified as the main vector [179–182]. These studies suggest that *Culicoides* species identified as vectors for BTV also act as vectors for the transmission of SBV [183, 184]. In Africa, little information exists on SBV presence in the livestock population as clinical and serological evidence of the virus have been provided only in four countries including South Africa, Mozambique, Tanzania and Nigeria [9, 185–187]. In the Nigerian study [9], SBV antibodies were detected in 29.2% of exotic and indigenous cattle tested using a commercial indirect ELISA kit that detects antibodies against recombinant SBV nucleoprotein in ruminant sera. However, in order to preclude the possibility of cross-reactivity with other Simbu serogroup viruses some of which have previously been reported in Nigeria, a larger serosurvey was conducted with cattle and sheep sera from different vegetation zones of the country using both the ELISA and serum neutralisation tests (unpublished observations). The authors detected serum neutralizing antibodies against SBV, Shamonda and Simbu viruses in cattle and sheep sera across the three vegetation zones studied.

### Akabane virus

Akabane virus (AKAV) is named after the Japanese village where the virus was first isolated from mosquitoes (*Aedes vexans* and *Culex tritaeniorhynchus*) in 1959 [188]. The virus belongs to the genus *Orthobunyavirus* of the family *Bunyaviridae*, in the Simbu serogroup of viruses [189]. Akabane virus is a teratogenic, *Culicoides*-borne virus that replicates in many kinds of natural host species and in several experimental animals [190]. Based on serological evidence, cattle, horses, donkeys, sheep, goats, pigs, camels and buffaloes appear to be infected in natural situations [191, 192]. AKAV has been shown to be an important pathogen causing seasonal epizootics of reproductive disorders (abortions, premature births, and stillbirths) and congenital arthrogryposis, hydranencephaly or microencephaly in cattle, sheep and goats, sometimes resulting in significant economic losses [5, 193].

In Africa, limited information based on virus isolation and/or serology revealed the presence of AKAV in Kenya [191, 194], Sudan [195, 196], South Africa [197, 198], Zimbabwe [199], Tanzania [187] and Nigeria [10]. In Nigeria, although abortions and congenital malformations associated with AKAV such as arthrogryposis, kyphosis, and scoliosis have been reported in ruminants [200, 201], the virus has not received adequate attention as a possible cause of these conditions. However, the recent detection of antibody-positive animals among unvaccinated cattle and sheep [10] provides evidence of AKAV infection in Nigeria. The findings highlight the need for further virological, entomological and molecular investigations in order to determine the role of AKAV as aetiology of abortions and congenital malformations in ruminants in Nigeria.

## Conclusions

Arthropod-borne viruses cause severe diseases of livestock resulting in great economic losses. Further, such infections often extend to the human population leading to morbidity and mortality with significant public health impact. In Nigeria, rapid urbanization coupled with the encroachment of individuals and their livestock into arthropod habitats has heightened the risks of animal to human transmission of viruses. However, despite these attendant risks and consequences of arboviral infections, the true economic and public health impact of these diseases are most likely underestimated, mainly due to under-reporting of disease events. Therefore, there is a need for increased awareness on the existence of these arboviral agents as well as continuous surveillance for them in order to facilitate their prompt identification and reporting to relevant government agencies. Routine serological, virological, entomological and molecular surveillance for these arboviral agents among livestock herds should be encouraged as their early detection would enable the deployment of pragmatic preventive measures to forestall their ‘escape’ into the human population. In addition, since the incidence of these diseases is generally higher during the rainy season due to increased vector activity, sentinel herd monitoring will yield useful data that can serve as the basis for an early warning and alert system to prevent future outbreaks.

## Abbreviations

AHSV: African horse sickness virus
AKAV: Akabane virus
ASFV: African swine fever virus
BEFV: Bovine ephemeral fever virus
BTV: Bluetongue virus
CCHFV: Crimean-Congo haemorrhagic fever virus
KOTV: Kotonkan virus
OIE: World Organisation for Animal Health
RVFV: Rift Valley fever virus
SBV: Schmallenberg virus
WAD: West African dwarf
WHO: World Health Organisation
WNV: West Nile virus
WSLV: Wesselsbron virus
